# Boosting Quantum Battery-Based IoT Gadgets via RF-Enabled Energy Harvesting †

**DOI:** 10.3390/s22145385

**Published:** 2022-07-19

**Authors:** Sumit Gautam, Sourabh Solanki, Shree Krishna Sharma, Symeon Chatzinotas, Björn Ottersten

**Affiliations:** 1 Department of Electrical Engineering, Indian Institute of Technology, Indore 453552, India; 2 Interdisciplinary Centre for Security, Reliability and Trust (SnT), University of Luxembourg, L-1855 Luxembourg, Luxembourg; shree.sharma@uni.lu (S.K.S.); symeon.chatzinotas@uni.lu (S.C.); bjorn.ottersten@uni.lu (B.O.)

**Keywords:** 5G and beyond/6G wireless networks, greencom, IoT, quantum battery, RF-energy harvesting, transmit power optimization

## Abstract

The search for a highly portable and efficient supply of energy to run small-scale wireless gadgets has captivated the human race for the past few years. As a part of this quest, the idea of realizing a Quantum battery (QB) seems promising. Like any other practically tractable system, the design of QBs also involve several critical challenges. The main problem in this context is to ensure a lossless environment pertaining to the closed-system design of the QB, which is extremely difficult to realize in practice. Herein, we model and optimize various aspects of a Radio-Frequency (RF) Energy Harvesting (EH)-assisted, QB-enabled Internet-of-Things (IoT) system. Several RF-EH modules (in the form of micro- or nano-meter-sized integrated circuits (ICs)) are placed in parallel at the IoT receiver device, and the overall correspondingly harvested energy helps the involved Quantum sources achieve the so-called quasi-stable state. Concretely, the Quantum sources absorb the energy of photons that are emitted by a photon-emitting device controlled by a micro-controller, which also manages the overall harvested energy from the RF-EH ICs. To investigate the considered framework, we first minimize the total transmit power under the constraints on overall harvested energy and the number of RF-EH ICs at the QB-enabled wireless IoT device. Next, we optimize the number of RF-EH ICs, subject to the constraints on total transmit power and overall harvested energy. Correspondingly, we obtain suitable analytical solutions to the above-mentioned problems, respectively, and also cross-validate them using a non-linear program solver. The effectiveness of the proposed technique is reported in the form of numerical results, which are both theoretical and simulations based, by taking a range of operating system parameters into account.

## 1. Introduction

### 1.1. Motivation

The world has consistently witnessed technological revolutions pertaining to Wireless Communications over the past few decades. Recent advancements in the field have narrowed down the gap between so-called binary information bits and the envisioned Quantum bits (Qubits). In this vein, the rapid transition to Quantum-based methods naturally demands an advanced framework that derives a technically synergistic relationship between the Quantum theories and Wireless-Communications-based techniques [1,2]. However, the main challenge in this context is our dependence on the energy sources, which is as strong as our dependence on the devices themselves [3,4,5,6].

Several works in the literature have focused on addressing these issues, and the interesting idea of a Quantum battery (QB) has been put forth by various researchers [7,8,9,10] in this context. More recently, the conceptual developments around the use of a nano-meter-sized QB has shown immense promise [11]. The working principle of the nano-meter-sized QB is similar to the phenomenon of Luminescence. Herein, nanostructured solid-state of matter in the nano-meter-sized QB works on the principle that the energy of photons can be transferred to the electrons present at the core of the QB, thereby increasing their lifetime (i.e., being in the energized state) to prolonged periods. The theoretical feasibility of this possibility is often determined by a quasi-stable state, which is primarily achievable within a lossless closed system. It is noteworthy that this phenomenon remains the backbone of several closed-system types of QBs [12]. Considering the present practical systems, it is almost impossible to ensure the lossless criterion for any closed-system design. Given that the persistent losses are bound to occur in the QBs, it could still be possible to utilize complementary systems to compensate for these losses, such as the use of radio-frequency (RF)-based wireless energy-harvesting (EH) techniques.

Nikola Tesla suggested an EH concept of extracting energy through the air during the late 1890s and early 1900s [13,14]. Since then, this concept has been applied to a variety of sectors of research, including wireless devices [15], flying objects [16], vehicles [17], and so on. Varshney recently explored the potential of adopting RF-based simultaneous wireless information and power transfer (SWIPT), which combines a standard information receiver with an EH module [18]. In this regard, various SWIPT receiver topologies, including time-switching (TS), power-splitting (PS), or separated architecture (SA), were investigated in the literature [19,20,21,22]. As such, the RF-based EH module is made up of several components, such as an RF-antenna, matching circuit, polarized capacitor, and rectifier circuit, and is hence considered a passive device [23]. The saturation effect in the diode element frequently causes problems for the EH module. As a result of the saturation effect, the non-linear nature of EH operation is noticed. This impact can be mitigated by connecting numerous EH circuits in a parallel manner, resulting in a linear region for operation [24,25]. The RF-based EH is also seen as a potential next step in the development of green wireless communication systems [26].

### 1.2. Current Studies and Related Works

Several studies have looked into the possibility of EH using ambient sources, i.e., thermal, mechanical, solar, and other sources, to power the QBs [27]. QBs using RF-EH mechanisms, on the other hand, are still an untapped and unexplored area of research. Due to the large size of the EH module, creating a system that integrates a QB and EH modules is difficult in the current scenario. Furthermore, the authors believe that, in the future, EH modules could be reduced to micro- to nano-meter-sized integrated circuits (ICs), which could be used to somehow aid QBs. The QBs require external help to compensate for any losses that may occur as a result of internal operations while maintaining their stability. As a result, a unique concept aimed at modeling and optimizing a design using a group of RF-EH ICs stacked in parallel to help a QB appears promising. The proposed approach could be used in Internet-of-Things (IoT) systems due to the small size of RF-EH-aided QBs. Quantum communications and QBs may also be explored, or used in conjunction in the IoT systems.

Regarding the practical or real-life scenarios, the framework proposed in this paper, which is based on the EH-enabled QBs, may be utilized in sensors, biomolecular analysis, photovoltaic devices, photochemical reagents, fluorescence, and light-emitting diodes (LEDs) [28]. When it comes to EH-assisted QB cores, the management of energy becomes crucial. In this regard, the frameworks and subsequent discussions provided in [29,30] tackle the main blocks used to manage energy in IoT nodes, which can further be translated towards management of the harvested energy for assisting the QB systems. In [31], the authors discussed power management in ultrasound-based EH in implantable medical devices, where the work is more focused on systems to manage power provided by an alternating current (AC) source (such as rectifiers and rectenna), and solutions for direct current (DC)-type sources are discussed in [32,33].

Most approaches concerning operation of QBs consider two-level operational frameworks (qubits), where a charger system assists in their promotion from the ground to excited state [9,34,35]. The charger system can be either quantum based or a classical time-dependent operation, driving the qubits [36,37]. In this vein, the key features of QBs are characterized in terms of energy storage and its maximum charging time [38]. In general, the characterization metrics, such as voltage, current, power and lifetime of QBs, can vary based on their types and operations. Correspondingly, the complexity of the circuit needed to sustain the QB operations may also be variable accordingly (e.g., utilization of micromasers as QBs [39]).

### 1.3. Novelty and Contributions

In this invited paper, we present a novel and futuristic framework considering an IoT system comprised of an RF-based transmitter and an end user equipped with a QB assisted by several RF-EH ICs. The RF-signals emitted by the transmit device are captured by a number of RF-EH ICs, where the overall harvested energy is then managed by a micro-controller. The micro-controller is also responsible for controlling a photon-emitting device. The photons dispatched from the photon-emitting device carry energy for the power-hungry electrons present at the core of a QB. In order to model and design the considered system, we formulate an optimization problem to minimize the overall transmitted power, subject to constraints on the overall harvested energy at the IoT-user node, and the number of RF-EH ICs. We investigate the problem in two ways. First, we analyze the problem using theoretical rigour to obtain the closed form solutions for the intended parameters. Second, we directly solve the (main) formulated non-convex problem using an optimization solver. Unlike [40], we formulate and solve an additional optimization problem to minimize the total number of RF-EH ICs, under constraints on the total transmit power and overall harvested energy (please note that this paper is an extension of the authors’ prior work, published as [40] (*©* 2021 IEEE)). Specifically, the main contributions and novelty of this work are summarized below.

1. We present a novel and futuristic model of an IoT-receiver device equipped with RF-EH ICs-assisted QB. In the proposed design, a QB is present at the core of the IoT-user device and is assisted via numerous RF-EH ICs.
2. In order to design and optimize this novel scheme, we formulate a couple of mathematical problems to minimize: (i) the overall transmitted power, subject to constraints on demanded harvested energy and number of RF-EH ICs; and (ii) the number of RF-EH ICs, subject to constraints on demanded harvested energy and total transmit power.
3. Correspondingly, we apply suitable convexification methods to find the optimal solutions to the above-mentioned problems. In this vein, we first obtain the closed-form solutions using theoretical analysis and then validate the results by directly solving the main (primal) problem via a non-convex optimization solver, sequentially, for both the problems.
4. We illustrate, with the help of numerical results, the benefits of the proposed design and related optimization of the considered framework. The outcomes motivate towards the realization of the proposed concept to a prospective real-life implementation.

The subsequent sections of this invited paper are organized as follows. Section 2 provides an introduction to the system model. The proposed RF-EH-assisted QB System design, and the problems corresponding to minimization of transmit power minimization and optimization of the number of RF-EH ICs at the IoT user, respectively, are presented in Section 3. Numerical results are shown and discussed in Section 4. Finally, concluding remarks are provided in Section 5.

## 2. System Model

We consider an IoT-based framework comprising a transmitter and an RF-EH ICs-assisted QB-enabled user, and discuss its possible prototyping and related challenges. Concerning the overall system design, we deliberate on some key aspects to provide adequate motivation for our proposed design. Acknowledging various possibilities of closed- and open-system models, several conceptualizations of QB designs have been proposed in the literature [7,8,9,10]. Owing to a wide range of evident challenges around the realization of open-system designs pertaining to the QBs [12], we therefore focus on the closed-system design of QB for our analysis.

**Figure 1:**
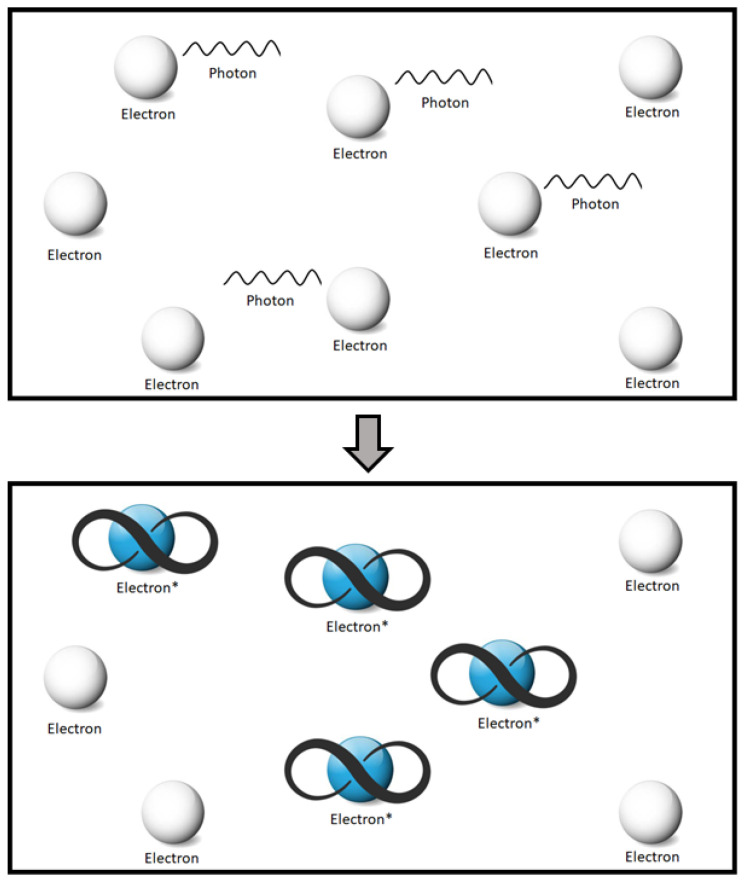
The phenomenon of the absorption of photons’ energy by the electrons in nano-structured solid state of matter within a closed system, where they may theoretically stay in this condition for extremely long periods of time [40].

Regarding the closed-system design, the notion of QBs broadly works on the principle of a photon being absorbed by an electron, which eventually reaches an excited state using the photon’s energy. Consequently, the excitation condition of the electron may prevail for extremely long periods of time. Based on this principle, conceptual nano-meter-sized QBs [11] have been proposed recently, which are proficient in holding the electric charge for a prolonged time period. The corresponding illustration of such a mechanism is depicted as in Figure 1. The above-mentioned principle also shares a similarity with the Luminescence effect, wherein certain goods, e.g., plastic objects, dials of watches, etc., glow in a dark environment for a very long time following the process of photon absorption.

It is noteworthy that, from a theoretical point of view, the above-mentioned process may seem sound and viable. However, it is extremely challenging to realize in practice given the wide range of influencing parameters, such as heat losses, environmental effects, losses pertaining to the respective functionalities of the components, etc. This makes it difficult to ensure a completely lossless environment, thereby leading to intractability, considering the current state of the technology. In this direction, there is now a growing interest around the design of QBs that can leverage from the harvested energy via ambient free sources of power, such as thermal, mechanical, and solar sources, etc., [27].

**Figure 2:**
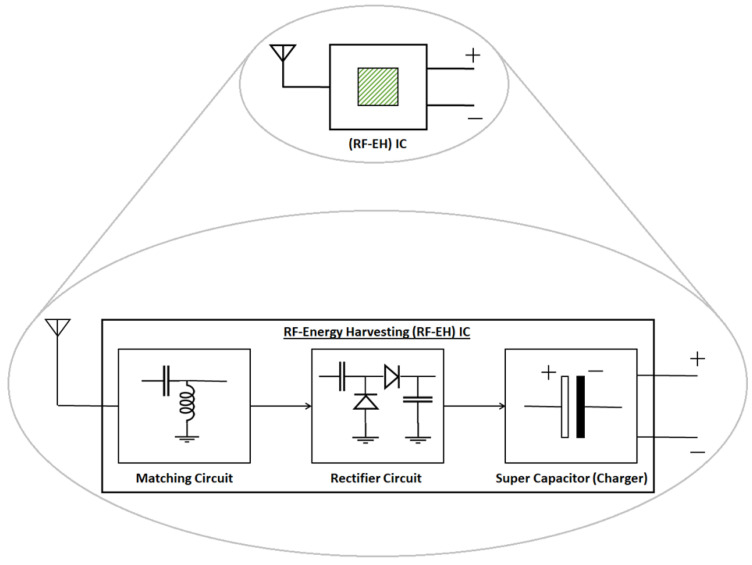
The miniaturization concept of RF-EH modules into micro- or nano-meter-sized ICs is depicted herein. As its working principle, an RF antenna absorbs the ambient (or specific band(s) of) RF signal and converts its energy into the equivalent DC type of output [40].

Harvesting energy via electromagnetic (EM) waves is another interesting prospect that has intrigued humankind for several years. In this regard, RF-based signals have been utilized for the purpose of EH to meet the needs of the battery-constrained wireless devices. Correspondingly, RF-based EH is yet another potential technique, wherein a passive elements-based matching circuit, a rectifier circuit and a charge-holding capacitor element constitute a basic EH module. Considering the current state of the art, the existing EH modules are constrained by large sizes, which are nearly equal to or greater than the dimensions of common handheld gadgets. The authors, however, envision that the EH modules will be miniaturized in future and can be made available in the form of RF-EH ICs, as shown in Figure 2. This motivation is backed by the fact supporting the evolution of RF-based information receivers, where the large sizes of modules have been/were drastically scaled down over time.

To this end, let us assume that the considered wireless RF-transmitter emits the signal x(t) via a single antenna, which is intended for the RF-EH IC(s). Noticeably, the benefits of using different waveforms to improve the EH efficiency may also be explored and exploited [41]. More specifically, a variety of waveform designs have been investigated and presented in the literature. However, it is difficult to comment on the best waveform that improves EH significantly, since it depends on the type of employed EH circuit. As a generalized assumption, we consider zero mean and unit variance signal in this invited paper to ensure a theoretical tractability.

Let us consider and denote the overall transmit power as $P_{T}$. The received signal at the QB-equipped IoT user is then given by: $y\left(t\right)=h\left(t\right)*x\left(t\right)+n_{R}\left(t\right)$, where h(t) is the complex channel gain coefficient for the wireless channel and $n_{R}\left(t\right)$ is the additive zero mean Gaussian noise at the corresponding IoT user’s receiving antenna equipment, with a noise variance of $\sigma_{R}^{2}$. Correspondingly, in case a linear EH operation is considered at the RF-EH IC unit of the receiver, then the relevant expression in this context is given as: $E^{L}=\zeta_{i}\left(P_{T}{\left|h\right|}^{2}+\sigma_{R}^{2}\right)$, where $0<\zeta_{i}\leq 1$ is the energy conversion efficiency of the corresponding receiver. In practice, we note that $E^{L}$ is analytically valid for theoretical evaluations; however, its real-time implementation is dubious. Therefore, we adopt the sigmoidal-function-based non-linear EH model [42,43,44] to characterize the EH operation, defined as

(1) $$E^{N}=\frac{E^{\prime }}{1-\phi }\cdot \left(\frac{1}{1+e^{\left(-\alpha P_{T}|h{|}^{2}+\alpha \beta \right)}}-\phi \right),$$

where $\phi \overset{\Delta }{=}\frac{1}{1+\exp(\alpha \beta )}$, the constant $E^{\prime }$ is obtained by determining the maximum harvested energy on the saturation of the EH circuit, and α and β are specific to the capacitor and diode turn-on voltage metrics at the EH circuit. For further analysis, we consider normalized time slots to use the terms power and energy interchangeably.

## 3. Proposed Design of RF-EH-Assisted QB System

**Figure 3:**
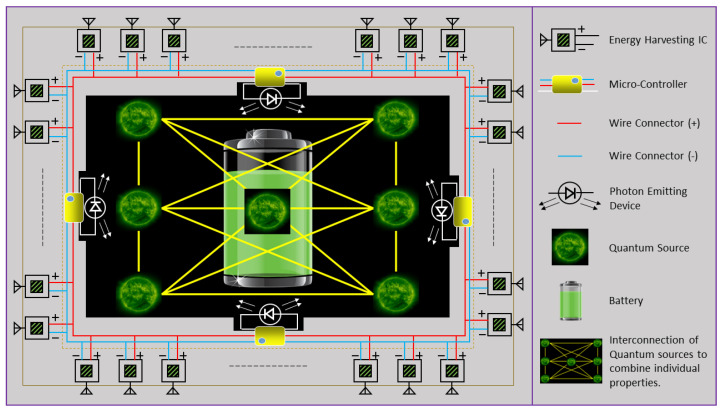
Prototype of the proposed novel and futuristic RF-EH ICs-assisted QB system [40].

Based on the concepts discussed and developed in the previous section, we present herein the proposed novel and futuristic model of an IoT system comprising a transmitter and QB-enabled end user assisted by numerous RF-EH ICs. The basic schematic of the proposed RF-EH ICs-assisted QB framework is shown in Figure 3, where the QB is assumed to be present at the core of the IoT user. The complete battery system is comprised of multiple RF-EH ICs, micro-controllers, photon-emitting devices, and the QB at the core. The RF-EH ICs are placed all across the system, such that the transmitted RF signal’s energy is extracted and then managed by the micro-controllers placed in different zones. The accumulated energy is then utilized by the micro-controllers to power the correspondingly connected photon-emitting devices, which thereby ensure the quasi-stability at the core of the QB.

As discussed before, ensuring a closed-system design in any QB is challenging, and hence, some losses are bound to occur in practice. However, these losses may be compensated by providing enough energy to the electrons present at the core of the QB. In this regard, the IoT-user device may contact the nearest transmitter, which may occur through regular RF-based Wireless Communications or via Quantum-based wireless communications systems using entanglement of Qubits [1,2]. The transmit source then uses RF signaling to transfer the demanded energy to the IoT user, where the energy is harvested with the help of multiple RF-EH ICs arranged all around the receive unit (it is explicit that the saturation effect of the diode prohibits a single unit of RF-EH IC from harvesting energy beyond a certain limit, and hence, multiple RF-EH ICs are assumed to be placed in parallel to overcome this issue). For our analysis, we consider that *n* number of RF-EH ICs are available, with a maximum cap of *N*. The selection of *N* is naturally decided according to the design requirements, e.g., the size of the device.

Upon the reception of the RF signal emitted by the transmit source, each RF-EH IC at the IoT user converts the signal energy using its miniaturized passive circuit. The management of the overall harvested energy is performed by a micro-controller. To emulate a lossless criterion corresponding to the closed-system design of the QB, the micro-controller is also responsible for the operation of an extremely low-intensity photon-emitting device (e.g., light-emitting diode (LED)), wherein the emitted photons facilitate the energy-transfer process at the core of the QB. The overall harvested energy demand at the IoT user is assumed to be ξ=η+κ. Herein, `η’ is the harvested energy demand for enabling the photon emission process. The additional `κ’ represents the harvested energy demand required for other consumption metrics, such as operation of the micro-controller, compensation for losses, and maintaining a suitable temperature within the closed system of the QB.

To proceed, we intend to model and optimize the involved metrics and parameters in the considered framework. Concretely, we first develop the formulation to minimize the overall transmitted power under limitations on the minimum harvested energy and the number of RF-EH ICs. Next, we formulate and solve another problem to optimize the number of RF-EH ICs at the IoT user, subject to constraints on the overall harvested energy demand and a maximum limitation on the transmit power. The following sections provide more insight into the considered problems and the obtained (proposed) solutions.

### 3.1. Transmit Power Minimization

In this section, we minimize the total transmit power, subject to the constraints on the overall energy harvested by the RF-EH ICs, and the number of (non-linear) EH ICs. The corresponding optimization problem can then be represented in its analytical form, as follows

(2) $$\begin{array}{ccc} \;\left(P1\right):\min_{n,P_{T}} & & \;P_{T}\; \end{array}$$

(3) $$\begin{array}{ccc} \;s.t. & & \;\left(C1\right):nE^{N}\geq \xi ,\; \end{array}$$

(4) $$\begin{array}{ccc} & & \;(C2):n\leq N.\; \end{array}$$

We find that the joint optimization of *n* and $P_{T}$ in (3) makes (P1) non-convex and intractable. However, an analytical solution based on a graphical/direct-evaluation method is possible, wherein one parameter may be relaxed/fixed to solve for the other, and vice versa. In this context, let us first observe that the objective in (P1) is independent of *n*. Next, we note that $E^{N}$ is an increasing function of $P_{T}$. Moreover, note that the value of either *n* or $P_{T}$ may be increased to satisfy the constraint in (C1). Concretely, the optimized solution of $P_{T}$ may be obtained by enforcing n=N, meaning that the equality in (4) must hold. In this vein, we leverage from this solution, i.e., n=N, to obtain/represent the the following (reduced) problem.

(5) $$\begin{array}{ccc} \;\left(P2\right):\min_{P_{T}} & & \;P_{T}\; \end{array}$$

(6) $$\begin{array}{ccc} \;s.t. & & \;\left(C1\right):NE^{N}\geq \xi . \end{array}$$

Note that (P2) is of a convex form, which implies that it should be possible to obtain either a closed-form solution or that the problem may be solved directly via well-known convex optimization methods [45]. Let us consider the former case, wherein we seek a suitable closed-form solution for $P_{T}$. To proceed, we observe that an optimal solution is achievable by letting the equality in (6) hold. This notion is based on the fact that the overall harvested energy demand at the IoT user will be sufficed by utilizing a minimized transmit power, provided that all the considered *N* RF-EH ICs are active. Therefore, we obtain a valid solution of $P_{T}$ based on the analysis mentioned above, expressed as follows (a detailed solution is provided in Appendix A; please refer to the same for more insights).

(7) $$P_{T}=\frac{1}{{\alpha |h|}^{2}}\left(\alpha \beta -\ln\left(\frac{NE^{\prime }-\xi \left(1-\phi \right)-N\phi E^{\prime }}{\xi \left(1-\phi \right)+N\phi E^{\prime }}\right)\right).$$

Hereby, we provide Algorithm 1 for the minimization of transmit power.
**Algorithm 1 **Minimization of Transmit Power.1:**Initialize**: *n*, $N=N_{\mathrm{Max}}$, $P_{T}=P_{\mathrm{Max}}$, ξ, α, and β.2:**Generate Channel Coefficients**: $h_{i}$, $i=\{1,2,\cdots ,M_{\mathrm{Max}}\}$, where $M_{\mathrm{Max}}$ is the upper limit for Monte Carlo experiment runs.3:**For theoretical validation**: Compute $P_{T}$ according to (7), keeping $n=N_{\mathrm{Max}}$.4:**For simulation validation**: Compute $P_{T}$ based on optimization via an interior point algorithm using fmincon(·) function in MATLAB, $\forall h_{i}$5:**To obtain different results (averaged over $M_{\mathrm{Max}}$)**: Keep varying values of $N_{\mathrm{Max}}$, $P_{\mathrm{Max}}$, and ξ.

Based on our analysis, it is clear that *N* plays a crucial role in the system design. An intuitive interpretation from the solution provided in (7) would be that the higher the number of RF-EH ICs in the device, the lower the transmitted power. As discussed before, the micro-controller can then manage the harvested energy to cater to the various needs to maintain the stability of the QB. In the following section, we present another optimization problem to optimize the number of RF-EH ICs, under the constraint on the overall harvested energy demand and the maximum transmit power limitation.

### 3.2. Optimization of the Number of RF-EH ICs

In this section, we optimize the total number of RF-EH ICs, subject to the constraints on the harvested energy by RF-EH ICs, and a limitation on the total transmit power. The corresponding optimization problem is represented in its mathematical form, as follows

(8) $$\begin{array}{ccc} \;\left(P3\right):\min_{n,P_{T}} & & \;n\; \end{array}$$

(9) $$\begin{array}{ccc} \;s.t. & & \;\left(C1\right):nE^{N}\geq \xi ,\; \end{array}$$

(10) $$\begin{array}{ccc} & & \;\left(C2\right):P_{T}\leq P_{\mathrm{Max}}.\; \end{array}$$

We note that (P3) is a non-convex problem, primarily due to the joint optimization of *n* and $P_{T}$ in (9). Similar to before, an analytical solution based on a graphical/direct-evaluation method is possible. Correspondingly, it is observed that the objective in (P3) is independent of $P_{T}$. In addition, we note that $E^{N}$ is an increasing function of $P_{T}$, while the presence of *n* in (C1) indicates that the constraint may be satisfied by either increasing its value or the value of $P_{T}$, as discussed in the previous section. Concretely, it becomes natural to choose the upper limit (value) of $P_{T}$, for the optimization of *n* as the objective of (P3). This translates to an implication that the equality in (10) must hold. Therefore, by leveraging from one solution, i.e., $P_{T}=P_{\mathrm{Max}}$, we reach the following (reduced) problem.

(11) $$\begin{array}{ccc} \;\left(P4\right):\min_{n} & & \;n\; \end{array}$$

(12) $$\begin{array}{ccc} \;s.t. & & \;\left(C1\right):n\cdot \frac{E^{\prime }}{1-\phi }\cdot \left(\frac{1}{1+e^{\left(-\alpha P_{\mathrm{Max}}|h{|}^{2}+\alpha \beta \right)}}-\phi \right)\geq \xi . \end{array}$$

Upon obtaining the convexified form (P4), we are now in a position to seek a closed-form solution or solve it via well-known convex optimization techniques [45]. To obtain a closed-form solution for *n*, we observe that an optimum solution is obtained when the equality in (12) holds. This is based on the fact that each RF-EH IC would be operational at the maximum possible limit (saturation regime). Hence, we turn to the following solution of *n* to address the saturation concern, based on the above-mentioned analysis (for a detailed solution, refer to Appendix B).    

(13) $$n=\frac{\xi (1-\phi )}{E^{\prime }\left(\frac{1}{1+e^{\left(-\alpha P_{{}_{\mathrm{Max}}}|h{|}^{2}+\alpha \beta \right)}}-\phi \right)}.$$

In the following, Algorithm 2 describes the steps to minimize the number of RF-EH ICs.
**Algorithm 2** Minimization of Number of RF-EH ICs.1:**Initialize**: *n*, $N=N_{\mathrm{Max}}$, $P_{T}=P_{\mathrm{Max}}$, ξ, α, and β.2:**Generate Channel Coefficients**: $h_{i}$, $i=\{1,2,\cdots ,M_{\mathrm{Max}}\}$, where $M_{\mathrm{Max}}$ is the upper limit for Monte Carlo experiment runs.3:**For theoretical validation**: Compute *n* according to (13), keeping $P_{T}=P_{\mathrm{Max}}$.4:**For simulation validation**: Compute *n* based on optimization via interior point algorithm using fmincon(·) function in MATLAB, $\forall h_{i}$5:**To obtain different results (averaged over $M_{\mathrm{Max}}$)**: Keep varying values of $N_{\mathrm{Max}}$, $P_{\mathrm{Max}}$, and ξ.

It is noteworthy that $P_{{}_{\mathrm{Max}}}$ plays a crucial role in the system design. An intuitive interpretation from the solution provided in (13) would be that the higher the transmitted power of the system, the lower the number of RF-EH ICs. Based on the optimized number of RF-EH ICs, the micro-controller can then manage the harvested energy to cater to the various needs to maintain the stability of QB. In the subsequent section, we present some simulation results to test the effectiveness of the proposed design in terms of the obtained solutions.

## 4. Simulation Results

Herein, we evaluate the performance of the proposed framework in terms of the minimized transmit power and the optimized number of RF-EH ICs, as obtained by solving the concerned mathematical problems discussed in the previous section. Direct implementation of the closed-form expressions are performed with the help of MATLAB R2019b, and the relevant closed-form solutions are referred to as `Theoretical’. Concerning the non-linear program solver, we make use of the *fmincon(·)* solver present in the optimization toolbox of MATLAB R2019b [46]. The corresponding outcomes are hereby described as `Simulation’ for further reference.

We assume an ITU-R outdoor framework (site-general model for propagation within street canyons) [47] to generate channel realizations with the path-loss exponent:

(14) $$PL\left(D,F\right)=10a\log_{10}\left(D\right)+b+10c\log_{10}\left(F\right)+N\left(0,\sigma \right)\;\mathrm{dB},$$

where *D* is the 3D direct distance between the transmitting and receiving stations (m), *F* is the operating frequency (GHz); the coefficients *a*, *b*, and *c* are associated with the increase in the path loss with distance, the offset value of the path loss, and the increase in the path loss with frequency, respectively; and N(0,σ) is a zero mean Gaussian random variable with a standard deviation σ (dB). The channel coefficients for the link between the transmitter and IoT user are generated accordingly. Specifically, we choose: *F* = 24 GHz; *D* is assumed to be 5 m (unless specified otherwise) for the IoT user with respect to the transmitter. *a* = 2.12, *b* = 29.2, *c* = 2.11 and σ = 5.06 dB (the value of *F* is chosen according to frequency range 2 (FR2) pertaining to the 5G-and-beyond systems [22]. Concerning the EH process via RF, the proposed method can be applied to other kind of sources and for different (more) scaled frequencies, such as frequency in the FM or AM bands or Bluetooth low energy (BLE) range (2.4 GHz) [48,49]). The constants corresponding to the non-linear EH circuit are chosen as $E^{\prime }$ = 2.8 *m*J, α = 1500, and β = 0.0022 [42,43,44]. Concerning every experimental run, we consider an average of 500 random channel realizations. For proper distinction among the closed-form and simulations-based solutions (obtained via *fmincon(·)* solver), we refer to the former as theoretical and the latter as simulation results. Table 1 illustrates the parameter value selections pertaining to the simulation environment.

**Figure 4:**
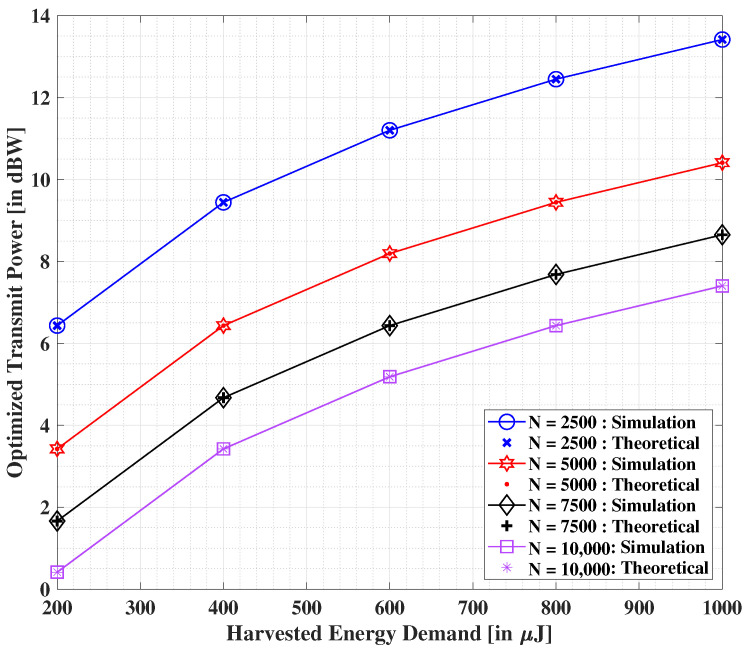
Minimized transmit power (in dBW) versus the demanded harvested energy for altering values of *N* [40].

In Figure 4, we illustrate the effect on the optimized transmit power values with the growing demand of harvested energy at the IoT user. Both the proposed theoretical and simulation-based solutions are compared herein. A non-linear increment is observed in the optimized transmit power with the escalating demand of harvested energy. On the other hand, when the number of RF-EH ICs is incremented steadily, we observe a considerable depletion in the corresponding values of optimized transmit power. The obtained results show that both theoretical and simulations-based solutions are more or less the same. Additionally, it is noted that the outcomes are in line with our intuitive analysis.

**Figure 5:**
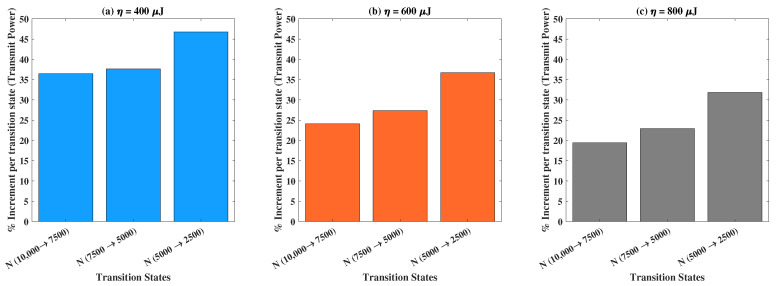
Percentage increment in transmit power versus different transition states pertaining to *N* for: (**a**) η=400 μJ, (**b**) η=600 μJ, and (**c**) η=800 μJ.

Using Figure 5, we will now discuss the percentage increase in the transmit power for various transiting states concerning the number of RF-EH ICs. The transit states are defined as the jump from one value of *N* to another, which determines our experimental outcomes. For example, N(10,000→7500) indicates the outcome when N=10,000 is transited to N=7500 for a fixed value of harvested energy demand. The corresponding percentage increment is defined by: $\%\;\mathrm{increment}=\frac{\mathrm{Final}\;\mathrm{Value}-\mathrm{Starting}\;\mathrm{Value}}{\left|\mathrm{Starting}\;\mathrm{Value}\right|}\times 100$. We consider three cases, vis-á-vis (a) η=400 μJ, (b) η=600 μJ, and (c) η=800 μJ, to investigate the percentage increment in the transmit power for various transiting states of *N*. The escalation in the percentage increment of transmit power from one state to another (in a successive manner) is noteworthy for each of the cases, as shown. The overall results imply the non-linear incremental relationship of the transmit power with the number of RF-EH ICs, for increasing values of harvested energy demands.

**Figure 6:**
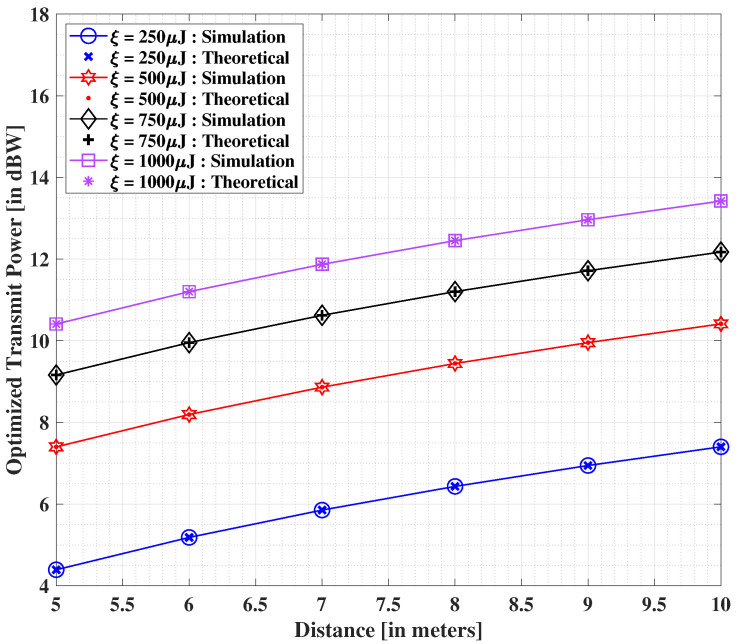
Minimized transmit power (in dBW) versus the demanded harvested energy, with N=5000 [40].

Figure 6 depicts the results corresponding to the impact of increasing distance values between the transmit source and the end user on the optimized transmit power, while keeping N=5000. As above, we compare the theoretical and simulation-based outcomes and find an identical nature concerning both the yielded solutions. As the distance between the transmitter and the IoT user increases, we see corresponding (significant) increments to the minimum transmit power values. It is additionally noteworthy that the above-mentioned effect amplifies with growing demands of harvested energy at a higher level. From the reported outcomes, the distance limitation pertaining to the EH mechanism is explicit.

**Figure 7:**
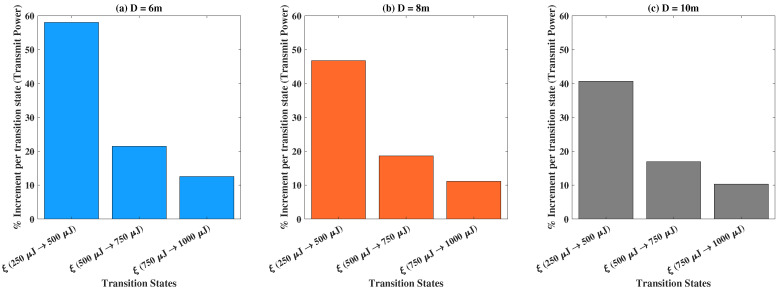
Percentage increment in transmit power versus different transition states pertaining to ξ for: (**a**) D=6 m, (**b**) D=8 m, and (**c**) D=10 m.

In Figure 7, we present the bar plots of the percentage increase in the transmit power for various transiting states pertaining to the harvested energy demands. Herein, we let transit states represent the move from one value of ξ to another consecutive value, which regulates the experimental outcomes. Yet again, e.g., ξ(250 μJ →500 μJ), implies the transit of the outcomes from ξ=250 μJ to ξ=500 μJ. The corresponding percentage increment in the transmit power is computed according to the definition provided previously. We compare the three cases: (a) D=6 m, (b) D=8 m, and (c) D=10 m, to find the percentage increment in the transmit power for various transiting states of ξ. Additionally, it is important to note the relative nature of percentages while examining two cases concerning *D* together. A depreciation in the percentage increment of the transmit powers is noted for the transits between the successive states for all cases. The relative outcomes indicate an incremental relationship of the transmit power with the harvested energy demands for appreciating values of *D*, while observing each transiting state individually.

**Figure 8:**
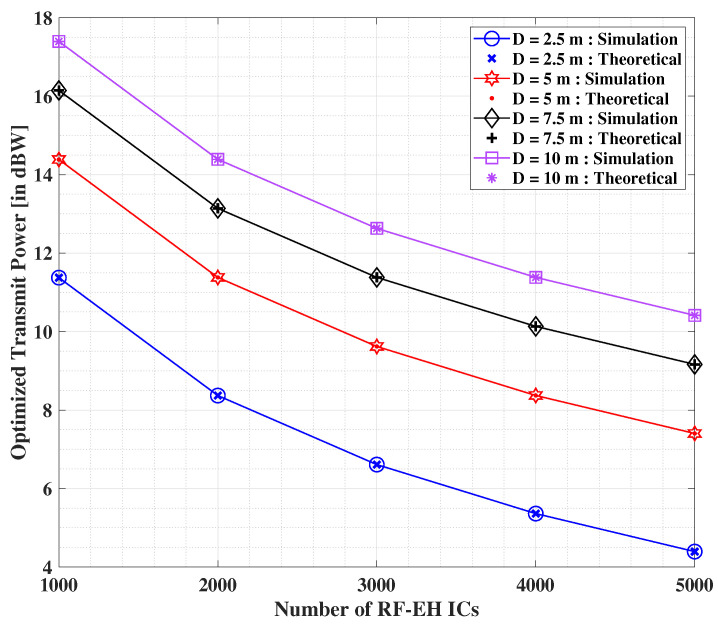
Minimized transmit power (in dBW) versus *N* for different value selections pertaining to the separation distance between the transmitter and IoT user [40].

The results shown in Figure 8 unveil the effect on the minimized transmit power values by increasing the number of RF-EH ICs, while assuming that ξ=500 μJ. We find again that both the analytical and simulation results are identical in nature, thereby implying the optimality of the theoretical outcomes. Herein, we observe a swift decrement in the values of the optimized transmit power in contrast with the growing values of *N*. In addition, a significant impact on this pattern is seen when the distance between the transmit source and the IoT user is increased, wherein an increment in the minimized transmit power values is reported. Nonetheless, it is needless to conclude that the transmit power optimization will be significantly better when the RF-EH ICs present at the IoT user are placed very close to the transmitter. Moreover, the results imply that a large enough number of RF-EH ICs at the IoT user would further ensure that the transmit power is minimized by a considerable amount.

**Figure 9:**
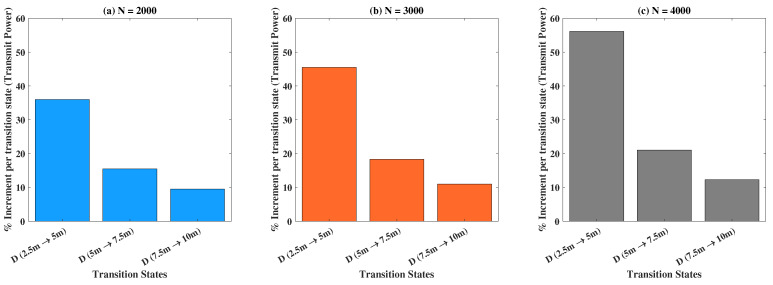
Percentage increment in transmit power versus different transition states pertaining to *D* for: (**a**) N=2000, (**b**) N=3000, and (**c**) N=4000.

We depict in Figure 9 that the bar plots of the percentage increase in the transmit power for various transiting states pertaining to *D*. The move from one value of *D* to another (consecutive) value is defined as the transit state in this case. More specifically, the notation D(2.5m→5m) implies that the starting point of the experiment is the optimized transmit power at D=2.5 m, the final value is obtained at D=5 m, and so on. Again, the percentage increment in the transmit power is computed as per the definition stated above. Herein, we compare the results corresponding to: (a) N=2000, (b) N=3000, and (c) N=4000 to find the percentage increment in the transmit power for various transiting states of *D*. Note that the percentage values are relative in nature for any two transition states or corresponding cases. This is derived from the results obtained in Figure 8, wherein the higher values of transmit power are seen to be at higher levels for D>2.5 m, while the increment in power values corresponding to D(5m→7.5m) and D(7.5m→10m) is seen to be marginal in comparison to D(2.5m→5m). Therefore, the depreciation in the percentage increase in the transmit power per transit state for the three considered cases is justified. The overall relative results indicate an incremental non-linear relationship of the transmit power, with respect to *D*, for increasing values of *N*, while observing each transiting state individually.

**Figure 10:**
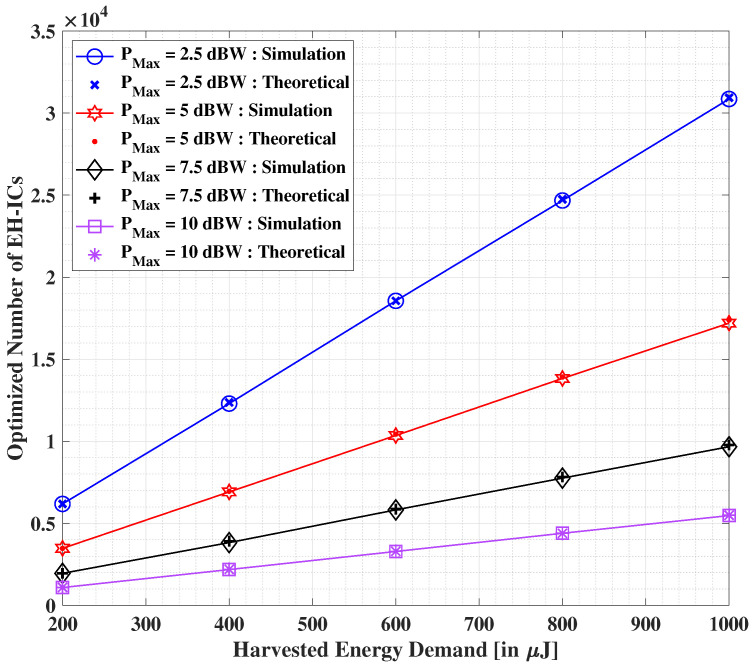
Optimized number of RF-EH ICs versus the overall demanded harvested energy demand for altering values of $P_{\mathrm{Max}}$.

Next, in Figure 10, we illustrate the variation in the optimized number of RF-EH ICs with the increasing demand of harvested energy at the IoT user. Similar to before, we compare the similarities between the outcomes pertaining to the proposed theoretical and simulation-based solutions. Herein, we observe that the optimized number of RF-EH ICs increases non-linearly with growing demands of harvested energy. However, a considerable decrement in the values of the optimized number of RF-EH ICs is seen with the increasing values of total transmit power $P_{\mathrm{Max}}$. We can note that both theoretical and simulation-based solutions yield similar results. It is noteworthy that these results seem to be in line with our intuitive analysis.

**Figure 11:**
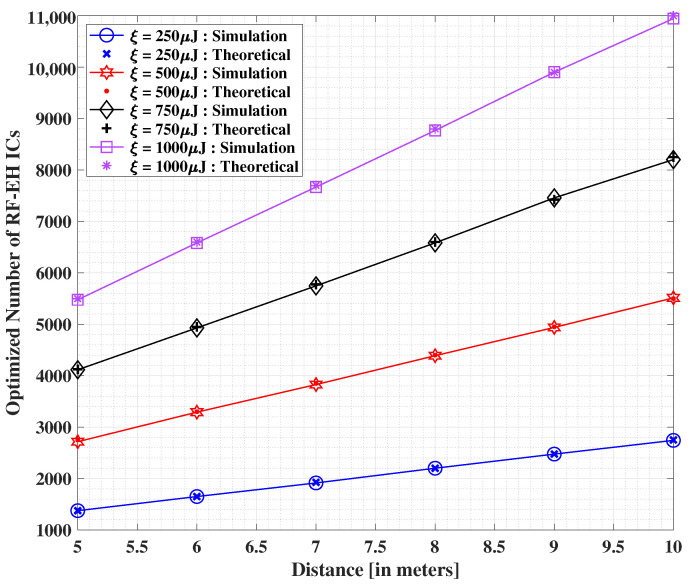
Optimized number of RF-EH ICs versus varying separation distances between the transmitter and end user, for different values of demanded harvested energy, where $P_{\mathrm{Max}}=10$ dBW.

In Figure 11, we show the effect of increasing the distance between the transmit source and the IoT user equipped with the QB on the optimized number of RF-EH ICs, while assuming $P_{\mathrm{Max}}=10$ dBW. Herein, we draw a comparison between the theoretical and simulation-based solutions, wherein both the solutions are reported to be almost identical. We observe that the minimized number of RF-EH ICs escalates with increasing distance values between the transmitter and IoT user. However, this effect amplifies at a higher level with the growing demands of overall harvested energy. The clear impact of distance limitation may be inferred herein, as seen in the previous results.

**Figure 12:**
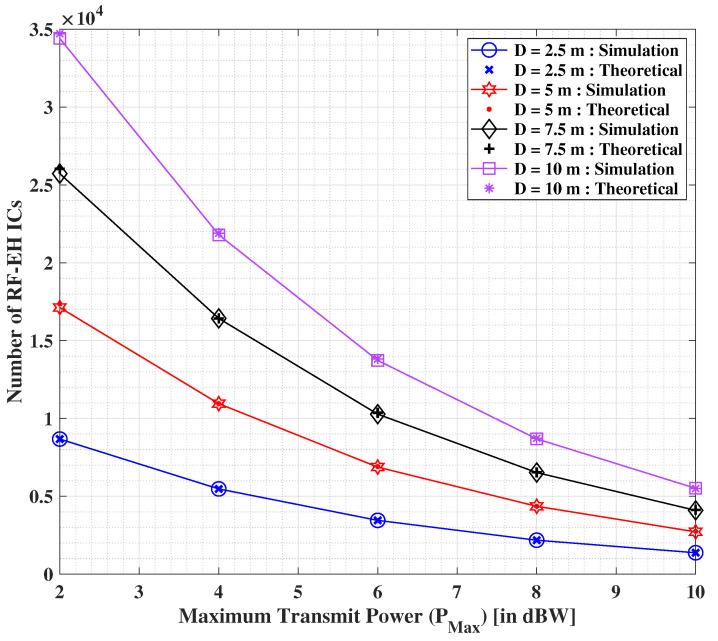
Optimized number of RF-EH ICs versus $P_{\mathrm{Max}}$ for altering selections of the separation distance between the transmitter and IoT user.

In Figure 12, we depict the results showing the impact of increasing the transmit power values on the optimized number of RF-EH ICs, with an assumption of ξ=500 μJ. Both the theoretical and simulation-based solutions are found to be more or less identical in nature. We observe that the minimum optimized number of RF-EH ICs depreciates with the increasing values of $P_{\mathrm{Max}}$. We additionally observe that the optimization yield corresponding to the transmit power intensifies with the increasing values of distance between the transmitter and the IoT user. This outcome suggest that the more closer the IoT user is to the transmitter, the better the optimization of the number of RF-EH ICs. Additionally, if the transmit power is large enough, the number of RF-EH ICs can be further minimized drastically. However, it is important to respect the maximum transmissible (allowable) power limitations deemed safe for living beings.

## 5. Conclusions

In this invited paper, we investigated a novel IoT system comprised of RF-EH ICs-assisted QB. A micro-controller was utilized to manage the overall harvested energy from several RF-EH ICs placed in parallel, and was also responsible for powering a photon-emitting device. The considered system was composed of a single transmitter and a single QB-enabled IoT user. In this vein, we firstly minimized the total transmitted power, subject to constraints on minimum number of RF-EH ICs, and harvested energy demand of the IoT user. Next, we optimized the number of RF-EH ICs subject to constraints on the total transmit power and harvested energy demands. For both the above-mentioned problems, we obtained suitable closed-form solutions and also cross-validated them using a non-convex optimization solver. Concerning future prospects, the outcomes from this work hold great promise in terms of both transmit power optimization while respecting the safety limits, and use of a minimal number of RF-EH ICs to maintain the stability of QB in a single IoT receiver unit. The proposed novel framework based on IoT systems equipped with RF-EH ICs-assisted QB may find applications in the domains where battery concerns are prevalent, specifically in compact devices with displays. Herein, the internal movement of electrons within a QB may be translated to a visual information via display, where this interesting idea can be taken up for future research. Some practical examples where RF-EH ICs assisted QB systems could come in handy are: electric automotives, wireless gadgets, biomedical sensors, and many other limited battery-constrained devices. In future, we plan to extend this work to a multi-user and multi-carrier scenario for the RF-EH process to improve the efficiency of the QB system.

## Figures and Tables

**Table 1:** Table for parameter value selections pertaining to the simulation environment.

Parameter	Value
*a*	2.12
*b*	29.2
*c*	2.11
σ	5.06 dB
$E^{\prime }$	2.8 *m*J
α	1500
β	0.0022
*F*	24 GHz
*D*	5 m (variable)

## Appendix A. Closed-Form Solution for the Transmit Power Minimization Problem

The non-linear EH constraint at IoT user, as in (6), is represented as

(A1) $$N\cdot \frac{E^{\prime }}{1-\phi }\cdot \left(\frac{1}{1+e^{\left(-\alpha P_{T}|h{|}^{2}+\alpha \beta \right)}}-\phi \right)\geq \xi ,$$

where ξ is the harvested energy demand at the IoT user.

We rearrange the expression in (A1) as

(A2) $$\frac{1}{1+e^{\left(-\alpha P_{T}|h{|}^{2}+\alpha \beta \right)}}\geq \xi \cdot \left(\frac{1-\phi }{NE^{\prime }}\right)+\phi ,$$

and rewrite it as

(A3) $$1+e^{\left(-\alpha P_{T}|h{|}^{2}+\alpha \beta \right)}\leq \frac{NE^{\prime }}{\xi \left(1-\phi \right)+\phi NE^{\prime }}.$$

The equation in (A3) implies that

(A4) $$e^{\left(-\alpha P_{T}|h{|}^{2}+\alpha \beta \right)}\leq \frac{NE^{\prime }-\left(\xi \left(1-\phi \right)+\phi NE^{\prime }\right)}{\xi \left(1-\phi \right)+\phi NE^{\prime }}.$$

Next, we take the natural logarithm on both sides of (A4)

(A5) $$-\alpha P_{T}{\left|h\right|}^{2}+\alpha \beta \leq \ln\left(\frac{NE^{\prime }-\left(\xi \left(1-\phi \right)+\phi NE^{\prime }\right)}{\xi \left(1-\phi \right)+\phi NE^{\prime }}\right).$$

An equivalent constraint on the transmit power is obtained upon further simplification of (A5), represented by

(A6) $$P_{T}\geq \frac{1}{{\alpha |h|}^{2}}\left(\alpha \beta -\ln\left(\frac{NE^{\prime }-\xi \left(1-\phi \right)-N\phi E^{\prime }}{\xi \left(1-\phi \right)+N\phi E^{\prime }}\right)\right).$$

From (A6), it is clear that in order to minimize the total transmit power, the equality in (A6) must hold. Therefore, we present the closed-form expression for $P_{T}$, as follows.

(A7) $$P_{T}=\frac{1}{{\alpha |h|}^{2}}\left(\alpha \beta -\ln\left(\frac{NE^{\prime }-\xi \left(1-\phi \right)-N\phi E^{\prime }}{\xi \left(1-\phi \right)+N\phi E^{\prime }}\right)\right).$$

QED.                                                              *■*

## Appendix B. Closed-Form Solution for the Optimal Number of RF-EH ICs

From (12), we have the following non-linear EH constraint at the IoT user

(A8) $$n\cdot \frac{E^{\prime }}{1-\phi }\cdot \left(\frac{1}{1+e^{\left(-\alpha P_{\mathrm{Max}}|h{|}^{2}+\alpha \beta \right)}}-\phi \right)\geq \xi ,$$

where ξ is the harvested energy demand at the IoT user, as before.

Rearrangement of (A8) leads to

(A9) $$n\cdot \frac{E^{\prime }}{1-\phi }\geq \xi \cdot {\left(\frac{1}{1+e^{\left(-\alpha P_{\mathrm{Max}}|h{|}^{2}+\alpha \beta \right)}}-\phi \right)}^{-1},$$

which can be further simplified as

(A10) $$n\geq \frac{\xi (1-\phi )}{E^{\prime }\left(\frac{1}{1+e^{\left(-\alpha P_{{}_{\mathrm{Max}}}|h{|}^{2}+\alpha \beta \right)}}-\phi \right)}.$$

From (A10), it is clear that in order to minimize the total number of RF-EH ICs, the equality in (A10) must hold. Therefore, we reach to the closed-form expression for *n*, as follows.

(A11) $$n=\frac{\xi (1-\phi )}{E^{\prime }\left(\frac{1}{1+e^{\left(-\alpha P_{{}_{\mathrm{Max}}}|h{|}^{2}+\alpha \beta \right)}}-\phi \right)}.$$

QED.                                                              *■*
